# The Care of Children's Teeth

**Published:** 1898-08

**Authors:** J. R. Lowe

**Affiliations:** Newton, Kas.


ARTICLE VI.

### THE CARE OF CHILDREN'S TEETH.

DR. J. R. LOWE, NEWTON, KAS.

Read before the Southern Kansas Dental Association, December 28th, 1897.

To determine what to do with the teeth of little children is ofter very perplexing to a conscientious dentist. Most children need our services often; and they need them badly. There is no decade in the life of a man or woman, no time "from the cradle to the grave," when the teeth need more painstaking care than from the age of three to thirteen years. We are inclined to suspect a disposition on the part of many in our profession to shirk their responsibility in this particular line of practice. The reasons for this are obvious. It requires much time and patience, taxes our nervous energies very severely, and is not always remunerative. We should remember, however, that the little boys and girls of to-day will be the young men and women of tomorrow, and if we successfully minister to their wants in childhood, they will appreciate us when they are older, and mothers, as a rule, are not slow to appreciate what we do for the little ones.

The question "What should be done with the teeth of children?" often resolves itself into one of "What can be done?" Much depends upon the tact of the operator and the disposition of the child. Some children are kind, confiding, and willing to do whatever we request of them; while others are by nature antagonistic, rebellious, and unmanageable except by force.

The president of the Illinois State Dental Association is reported to have said in his annual address last spring that a dentist should have the head of a scientist, the skill of a surgeon, the heart of a mother, and the touch of an angel. The last two qualifications are especially requisite when it comes to dealing with children.

"The American System of Dentistry," out of over three thousand pages, has just three pages relating to this subject. True, there is considerable space devoted to "diseases incident to the first dentition," but these usually within the domain of the medical practitioner.

When the child first comes under our care it is usually three or four years of age, the immediate occasion of the visit being a case of odontalgia, whereby the entire household has been kept awake a goodly portion of the preceding night. The parent demands immediate extracting, of course. Just here the dentist has a golden opportunity to impart some wholesome and much-needed advice. Fortunate for the child if he makes the best of the opportunity.

The first thing in order, of course, is the relief of pain. Beyond this but little should be attempted at the first visit, except to cultivate the child's acquaintance, get him interested, and put him in such a frame of mind that he will not be unwilling to come a second time. This should be the end of the first visit, as far as the child is concerned. How about the parent? If the dentist is of smooth address, and if he possesses a goodly fund of the information which "comes handy" on all such occasions, he can in a comparatively short time arouse the parents to a sense of their responsibility and send them away with much to reflect on pertaining to oral hygien, the importance of the deciduous teeth, the effect they have on the general health, the irregularities of the permanent set caused by injudicious extracting, and especially the time for the advent of the sixth year or first permanent molar. For whoever knew a parent who did not mistake this tooth for one of the deciduous set? A child has just twenty fingers and toes, and also just twenty temporary teeth. When this analogy in numbers has been pointed out to the parents, they will very likely remember it, and by counting will be enabled to know when teeth other than those of the deciduous set have made their appearance.

Notable among the exciting causes of caries in the teeth of children is the habit of eating at irregular times (between meals). When this habit prevails no attention is paid to keeping the teeth clean.

A great predisposing cause is the high pressure under which we live, the lack of sufficient outdoor life, imperfect digestion and consequent imperfect assimilation. The bicycle, however, has remedied this evil to a great extent. Many children start to school too young, and many lads and lasses have a notion, which is also shared by their parents, that their reputation for "smartness" will be forever established if it can only be said that they were quite young when they graduated from the high school or academy. How much more to their advantage will it be if, when they have attained mature age, it can be said of them that they possess sound minds in sound bod

Who will then know or care whether they "crossed the Alps into Italy" at the age of seventeen or at the age of twenty?

We have said that but little should be attempted at the child's first visit to the dentist beyond the relief of pain, interesting the little one, and getting his confidence. How can this be done? By a few rides up and down on the operating chair, by explaining the use of the mouth-mirror, initiating the patient into the mysteries of the dental engine, or any other method that may suggest itself. How can we keep a child's confidence? Tell him the truth; do not make promises you cannot fulfil. If the work to be done is going to cause slight pain, tell him frankly that it will hurt "just a little," and then see to it with religious scrupulosity that it is no more. By appealing to their pride we can usually induce them to bear slight pain, but severe pain is out of the question.

A comparatively recent volume, entitled "Diseases of Children's Teeth, Their Prevention and Treatment," by an English author, Richard Denison Pedley, has been published in England and America. It is addressed to to practitioners of dentistry and medicine, but more especially the latter. It offers many valuable suggestions. He recommends the application of nitrate of silver "in simple caries and where there is no great depth of decay." First wipes the tooth dry, winds a little cotton on the end of a stick, slightly moistens it with water, rubs it on the caustic and then on the decayed surface. It allays sensitiveness and arrests decay, but has one disadvantage, viz., that of blackening the teeth. In the anterior teeth he would use liniment of iodin, which allays sensitiveness, but does not arrest decay.

To relieve pain from inflamed pulp he uses "carbolized rosin," which consists of rosin one ounce, carbolic acid one ounce and chloroform half an ounce. He devitalizes pulps of deciduous teeth by repeated applications of this preparation. Condemns use of arsenic in deciduous teeth. Removes contents of pulp-chamber, but makes no effort to remove the pulp from the roots. Puts pledget of cotton dipt in iodoform or oil of cassia in pulp chamber, places a disk of card, cork or tin over it and fills with gutta-percha or amalgam. So far our author's doctrine appears wholesome and worthy of acceptation. But in the next paragraph he says that if the pulp is dead and the tooth discolored, to fill such a tooth would be dangerous. Says if it is firm in its socket and free from soreness, it should be cut down level with the gum and allowed to remain; but if it be loose and sore to bite on, it should be extracted an once. We would prefer to treat and fill such a tooth much the same as if it were in the mouth of an adult. All the above relates to the deciduous teeth, but the author recommends substantially the same procedure with a permanent molar, with a gangrenous pulp. This does not harmonize with the good sense which elsewhere characterizes this author's work, but sounds more like a voice from the dead, forgotten past—when the turnkey and carved ivory teeth held sway.

With what shall we fill children's teeth? My own practice has been to use principally amalgam and cement. Success, however, depends not so much on our choice of material as on our being able to prepare the cavities and exclude moisture while the filling is being inserted.—*Western Journal*

